# Reaching the Nadir of Medication Overuse in Chronic Migraine

**DOI:** 10.3390/ijerph192214696

**Published:** 2022-11-09

**Authors:** Dilara Onan, William David Wells-Gatnik, Paolo Martelletti

**Affiliations:** 1 Back and Neck Health Unit, Faculty of Physical Therapy and Rehabilitation, Hacettepe University, Ankara 06800, Turkey; 2 Department of Clinical and Molecular Medicine, Sapienza University, 00185 Rome, Italy; 3 Regional Referral Headache Center, Emergency Medicine Unit, Sant’Andrea University Hospital, 00189 Rome, Italy

**Keywords:** calcitonin gene related peptide, ditans, gepants, medication overuse, chronic migraine

## Abstract

The introduction of new drug classes for chronic migraine, such as monoclonal antibodies for calcitonin-gene-related peptide or its receptor (CGRPr), or antagonists of the same CGRP, have opened a new scenario in a selected population of individuals with migraine, and those presenting with chronic form of migraine in association with medication overuse. Medication overuse is now considered a complication of chronic migraine and, in fact, the treatment with CGRP(r)-MAbs of chronic migraine with medication overuse results in a clinical improvement of chronic migraine itself, accompanied by a parallel and obvious reduction in the intake of specific and non-specific acute migraine drugs. Education on the correct use of these drugs will be an essential tool to reduce the disability and costs of people suffering from CM complicated by MO, considering the long-term safety of the new therapies targeting the CGRP pathways. Only in this way can medication overuse risk can be reduced at its nadir in the scenario of chronicity of migraines.

## 1. Introduction

The therapy of acute migraine attacks has always encountered obstacles relating to safety, due to the onset of adverse cardiovascular or gastrointestinal events, to the exclusion criteria in the selection of potential patients, and to the progressive reduction in prescriptive adherence, given the natural evolution of migraine towards a numerical progression in terms of headache days and pain intensity scale. In the absence of adequate awareness towards people with migraine that reached the threshold of 4 headache days/month, it is necessary to counteract this evolution in chronicization by starting prevention/prophylaxis therapy to avoid moving towards the slippery slope of medication overuse (MO).

The introduction of new drug classes for chronic migraine (CM), such as monoclonal antibodies for CGRP(r) (CGRP(r)-MAbs) or antagonists of the same CGRP (CGRP(r)-AG), has opened a new scenario in a selected population of individuals with migraine, those presenting CM in association with MO, or with the even more dangerous risk of evolving into resistant or refractory CM [1,2]. The importance of MO in CM is evidenced by the tendency of these patients to frequent relapses, even after rehabilitation, pharmacological procedures, and psychological support [3,4], leaving the door open to an interpretation of the same addiction to substances [1]. The border between medication overuse and refractoriness to any therapy in chronic migraine is yet to be understood. The common ground of refractoriness is surely the high psychiatric comorbidity that some of these patients show, as well as the impossibility for a minority of them to have no benefits from any prophylaxis and/or detoxification procedures. For these reasons, it has been hypothesized that this deeper subset might be identified as drug dependence. In fact, often, they are users of barbiturates, opioids, and/or ergot derivatives (where still available) [5]. The high social and personal costs associated with the burden of CM itself have clearly indicated how the watershed of an effective and safe therapy can thus be removed by offering an adequate social, work-related, and economic recovery to the people affected by migraines [6]. The satisfaction of personal and collective unmet needs will be able to be achieved in most subjects suffering from CM also through the efficiency of the organizational assistance structures dedicated to this social disease [7]. This path will provide personal benefit and a significant psychosocial relapse that will help to reduce the enormous current healthcare expenditure necessary for the management of this huge number of individuals suffering from migraines. However, MO management cannot simply be solved by adding another medication, but needs to comprise a multi-disciplinary approach addressing lifestyle factors (e.g., sleep, exercise, stress coping, etc.), other headache comorbidities, psychological and medical comorbidities, pain physical therapy, and substance hyperuse. This widens the opportunity to have additional tools for the treatment of CM with MO. 

In addition, it must be said that these patients need continuous support, and not only to discourage the biobehavioral tendency towards compulsive self-medication, sometimes even carried out in prediction of a migraine crisis, which guarantees them the non-exclusion from the social circuit. Therefore, from these considerations, it emerges that the new pharmacological classes of prevention must be applied as an early interdiction to the chronic phase to express their full rehabilitation potential.

## 2. Medication Overuse Issue in Chronic Migraine

MO is now considered a complication of CM [1], and, in fact, the treatment with CGRP(r)-MAbs of CM with MO results in a clinical improvement of the CM itself, accompanied by a parallel and obvious reduction in the intake of specific and non-specific acute migraine drugs [8,9].

The concept of a single pathway (CGRP) for both preventive and acute therapy is entirely new in the therapeutic framework of headaches. For decades, the standard of care for acute migraine attacks has offered us a jeopardized therapeutic choice on different pathways of action, such as 5HT_1B/D_ for triptans and cyclooxygenase-1 (COX-1) for non-steroidal anti-inflammatory drugs (NSAIDs), creating potential drug–drug interactions (DDIs) and competitive metabolic destiny favoring adverse events (AEs). Therapeutic failure could be imputed to the prescriptions of drugs that were not properly metabolized in a specific patient. Furthermore, often, treatment failures lead the patients to an overuse of acute medication [10].

Reconsidering the migraine standard of care today, we must agree that despite the existence of a CGRP pathway for both acute and preventive management, there is not enough evidence supporting the use of Rimegepant acutely in patients taking Rimegepant as a preventative, as well as the fact that gepants and CGRP MoAbs do not share a single target, as they have significant pharmacokinetic and pharmacodynamic differences. 

Therefore, a reflection on a hypothesis of positioning the new preventative anti-CGRP(r)-Mabs [11], the CGRP receptor antagonists [12], and the only receptor 5-HT_1F_ agonist [13] was necessary in this new era of migraine treatment.

Although for the targeted oral CGRP prophylaxis, clinicians can also rely on the use of gepants (Atogepant and Rimegepant), they can also rely on the acute treatment of the same Rimegepant, as well as Ubrogepant, all with oral delivery, and, soon also, Zavegepant administered intranasally [14,15,16,17].

Therefore, over time, the targets 5-HT_1B/D_ (triptans) and cyclooxygenase-1 (COX1) (NSAIDs) will be gradually abandoned since CGRP, whose use in migraine prevention has not shown particular AEs or tachyphylaxis, as reported in randomized controlled trials. However, in real world settings CGRP targeting may worsen overall very high safety profile in randomized controlled trials. However, CGRP antagonism may worsen sepsis survival, wound healing, or cardiovascular protection.

It is important to evaluate over time the available critical clinical data that are appearing in the literature concerning the long-term safety for the use of gepants, especially in patients with cardiovascular diseases, and potential combination effects with other antimigraine treatments, especially in CM use [18]. 

The use of CGRP(r)-MAbs prophylaxis or CGRP receptor antagonists in CM complicated by MO will substantially reduce MO, especially in the 30–40% of patient non-responders to triptans [19,20]. 

Moreover, the European Headache Federation has just published the 2nd Edition of the Guidelines for the use of CGRP (r) MoAbs, placing this pharmacological class as the first choice in the migraine therapy process [21]. This will induce a virtuous circle in Europe, even in those countries where they are not yet available through the NHS (i.e., France) and it will certainly be an advantage for migraine sufferers. Early treatment together with lifestyle modification and appropriate co-treatment of medical comorbidities shoud be considered as a mean of intercepting the chronic phase and subsequent MO.

This path will progressively guarantee a path of control of chronic migraine and its complications, and the control of a pathology, migraine, which currently has a prevalence of 14% in the population worldwide [22] and of CM, which reaches 4.6%, and reducing the progression towards migraine refractoriness will together reduce the combination therapies that are often the main metabolic route for the establishment of MO itself [23,24,25], leading this enormous clinical problem to be no longer visible on the horizon of the patient with CM, or at least by reducing its visibility to their nadir.

## 3. Conclusions

Medication overuse is an important consequence of CM that develops in individuals predisposed to drug addiction. Medication overuse in CM has been dealt with, up to now, looking at its essence alone, even hypothesizing its autonomous entity.

Current pharmacological research has produced drugs that target CGRP, which show both in the prevention and in the management of the attack a complete reverse pattern action on MO in CM. In the next few years, we will see the progressive permeation into clinical medicine of these new drugs that will reduce the current epidemiology of CM, and, consequently, the complication of MO.

Education on the correct use of these drugs will be an essential tool to reduce the disability and costs of people suffering from CM complicated by MO.

Another mandatory need is also the continuous education of specialists, or general practitioners or emergency departments, who face the patient with CM and MO in different settings.

Finally, the pharmacist’s role is strategic in discouraging analgesic storage behaviors, or worse, combination drugs containing opioids, barbiturates or triptans typical of these patients.

The current availability of European Guidelines for the treatment of migraine with innovative drugs that have the CGRP as a preventive target requires an endorsement by the individual European NHS for a widespread application of prevention measures useful to eliminate or at least minimize this well-hidden social plague that is medication oversue.
